# Reliability of measurements of the fractured clavicle: a systematic review

**DOI:** 10.1186/s13643-017-0614-4

**Published:** 2017-11-03

**Authors:** Paul Hoogervorst, Gerjon Hannink, Arnoud R. van Geene, Albert van Kampen

**Affiliations:** 1 Department of Orthopedics, Radboud University Medical Center, P.O. Box 9101, 6500 HB Nijmegen, The Netherlands; 2 Onze Lieve Vrouwe Gasthuis, Amsterdam, The Netherlands

**Keywords:** Clavicle, Fracture, Imaging, Shortening, Reliability, Reproducibility

## Abstract

**Background:**

The objective of this systematic review was to evaluate the reliability and reproducibility of measurements of shortening in midshaft clavicle fractures (MSCF) using any available imaging technique.

**Methods:**

Electronic databases (PubMed, EMBASE, and Cochrane) were searched. The 4-point-scale COSMIN checklist was used to evaluate the methodological quality of studies.

**Results:**

Four studies on reliability of measurement of MSCF were identified. These studies were of fair and poor quality. The reported intrarater reliability varied between none to fair, and intrarater reliability was minimal.

**Conclusion:**

No definite conclusions could be drawn. In order to optimize future studies and the realization of comparable results, more research is necessary to identify a standardized method of imaging and measuring.

Level of Evidence III.

**Electronic supplementary material:**

The online version of this article (10.1186/s13643-017-0614-4) contains supplementary material, which is available to authorized users.

## Background

Fractures of the clavicle are common, comprising up to 5% of all fractures in adults [1]. Most clavicle fractures are localized at the level of the mid-diaphyseal third [2]. Dislocation of the fracture elements in midshaft clavicle fractures (MSCF) occurs due to the actions of the sternocleidomastoid muscle, which displaces the medial fragment superiorly and posteriorly, and of the deltoid and great pectoral muscles, which shift the lateral fragment inferiorly and anteriorly. These shifts cause a malaligned fracture that may result in symptomatic malunion of the clavicle and increase the risk of a nonunion [3–6].

In the last decades, many studies have reported that a shortened clavicle can lead to worse functional outcomes, pain, loss of strength, rapid fatigue, hyperesthesia of the hand and arm, difficulty sleeping on the affected side, and esthetic complications [5–14]. Godfrey et al. [15] reported that the degree of symptomatology and occurrence of mal- and nonunion after MSCF is related to the extent of shortening and displacement of the fracture elements. Mean post-traumatic shortening of the fractured clavicle has been reported to be approximately 1.2 cm; however, shortening of up to 3 cm has been reported [16]. It has been described that there are poorer outcomes when shortening of the clavicle is more than 15–20 mm or 9.7–15% as compared to the original length [5, 7–14].

For this reason, lately, the tendency has been to surgically reduce and fixate MSCF if shortened more than 15–20 mm, or if displaced more than the diameter of the clavicle’s shaft. However, due to the unique shape of the clavicle, consisting of an S-shape in two planes, reliable and reproducible measurements of the displacement and shortening can be challenging.

Although there is a plethora of available modalities and techniques to measure shortening of the MSCF, it still remains unclear which method is most accurate, reproducible, and useful in daily practice.

Therefore, the objective of this systematic review was to evaluate the reliability and reproducibility of measurements of shortening in MSCF using any available imaging technique.

## Methods

Electronic databases (PubMed, EMBASE, and Cochrane) were searched from their inception to November 2016. Keywords used to develop our search strategy were “clavicle,” “fractures,” “imaging,” “shortening,” “displacement,” and “reliability.” The detailed search strategy is described in Additional file 1. The inclusion criteria and method of analysis were specified in advance and documented in a protocol that was not registered in PROSPERO (Additional file 2).

### Inclusion criteria

All titles and abstracts were screened, and study inclusion was decided on by two reviewers (PH/GH). In case of discrepancy in study, inclusion disagreements were discussed until consensus on eligibility was reached. References of retrieved eligible articles were searched for supplementary studies. Studies meeting the following criteria were included:

- Studies aiming to assess shortening of the fractured clavicle for intrarater and interrater reliability.
- Studies investigating methods of imaging of the fractured clavicle for intrarater and interrater reliability.
- Only original studies were included.
- Studies in Dutch or English.
- Study population aged 9 years and older.

Abstracts, theses, and conference proceedings were not included.

### Data extraction and quality assessment

An electronic data extraction form was created and used to record data. Data from all included studies were extracted with respect to specific characteristics, that is, number of clavicles reviewed, study design, imaging technique, method of measurements, statistical analysis, and the author’s conclusion. PH and GH extracted data independently. If disagreement persisted after discussion, consensus was met consulting AvK.

Methods and quality were independently assessed (PH and GH, any discrepancies were discussed to achieve consensus, using a third reviewer (AvK) for all included studies. The 4-point scale COSMIN checklist box B for assessment of reliability was used.

The “worst score counts” algorithm was used for the analysis [17]. Briefly, each item from COSMIN box B was rated individually as “excellent,” “good,” “fair,” or “poor,” and an overall score was given by taking the lowest score of any of the items.

**Fig. 1:**
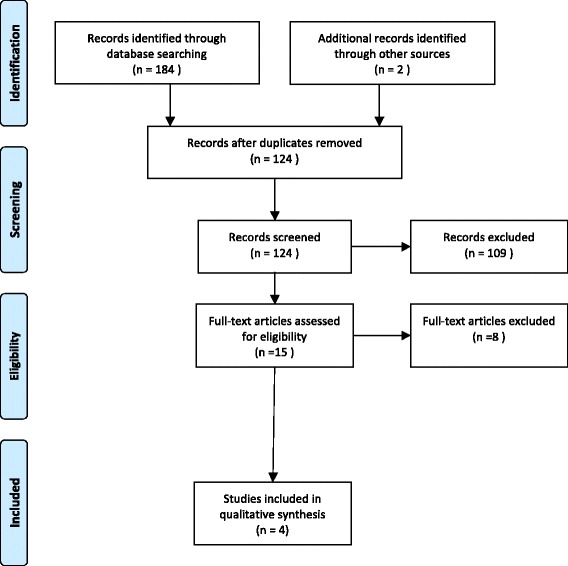
PRISMA flow diagram

The PRISMA (Preferred Reporting Items for Systematic Reviews and Meta-Analyses) guidelines, both the PRISMA flowchart and checklist, were followed during the preparation of this review (Fig. 1 and Additional file 3).

## Results

In total, 184 studies were identified. After the removal of duplicates, 122 studies were selected for the screening of titles and abstracts. Reference tracing and hand searching yielded two more possibly eligible studies. After the selection of titles and abstracts, 15 studies were selected for a full-text evaluation. After full-text evaluation, four studies were included in this systematic review and were used for data extraction (Fig. 1—flow diagram). Table 1 shows the extracted data of the four studies included in this systematic review.

**Table 1:** Extracted data included studies

Author	Number of patients	Goal of study	Modality	Measurements	Observers (role)/occasions	Statistics	Conclusion
Jones (2014) 18	30	Reliability Shortening in mm Displacement in % Comminuted Y/N Surgery Y/N	X-ray AP 30° caudo-cranial	Calibrated measuring tool	13 (13 surgeons) 2 (after 4 months)	Power analysis + intraobserver shortening 0.38 Interobserver shortening 0.33 *K* values stated	Strong inter- and intraobserver agreement for displacement and comminution
							Weak to no interobserver and minimal intraobserver agreement for shortening
Silva (2013) 19	32	Interobserver and intraobserver reliability shortening in MSCF in adolescents (9–18)	X-ray 15° caudo-cranial AP	1. Observers’ method of choice	7 (3 pediatric orthopedic surgeons, 1 pediatric orthopedic fellow, 2 orthopedic residents, 1 medical student) 2 (1 week)	1. Interrater reliability 0.771 and 0.743 Intrarater reliability 2.62	Standardized method of measuring not better than method of choice
				2. Standardized method Digital measurement system		2. Interrater reliability 0.741 and 0.685 Intrarater reliability 3.34	Interrater no significant difference
							Intrarater significant difference
Smekal (2008) 20	30	Assess different measuring methods and determine the most accurate method compared to CT	X-ray PA thorax 15° caudo-cranial AP of both clavicles 15° caudo-cranial AP clavicle CT	Standardized methods as described in article	4 (3 orthopedic surgeons, 1 radiologist) 2 (after 1 month)	Determination of differences of mean values: paired *t* test or nonparametric Wilcoxon signed-rank test	Differences among measurements on X-ray and CT not significant Repeatability coefficient on AP 15 Clavicle and clinical measurement low
						Distribution from: Kolmogorov-Smirnov test	Also smallest agreement with CT
						Repeatability: RC according to Bland-Altman A *p* < 0.05	PA thorax to determine length differences
Archer (2016) 21	22	Identify correlation between plain film and computed tomography (CT) measurement of displacement and the inter- and intraobserver reliability of repeated radiographic measurements	X-ray AP plain film CT	No standardized method of measurement	6 (3 orthopedic surgeons 3 residents)	Correlation using the Bland-Altman reliability coefficient Limits of agreement using the standard deviation (SD): 3.48 reliability using the Cronbach *α* coefficient. 0.90 Intraobserver reliability using paired *t* tests for each observer: all but one > 0.05	Plain film measurements of acute MSCF do not reliably predict shortening

### Methodological quality of the studies

**Fig. 2:**
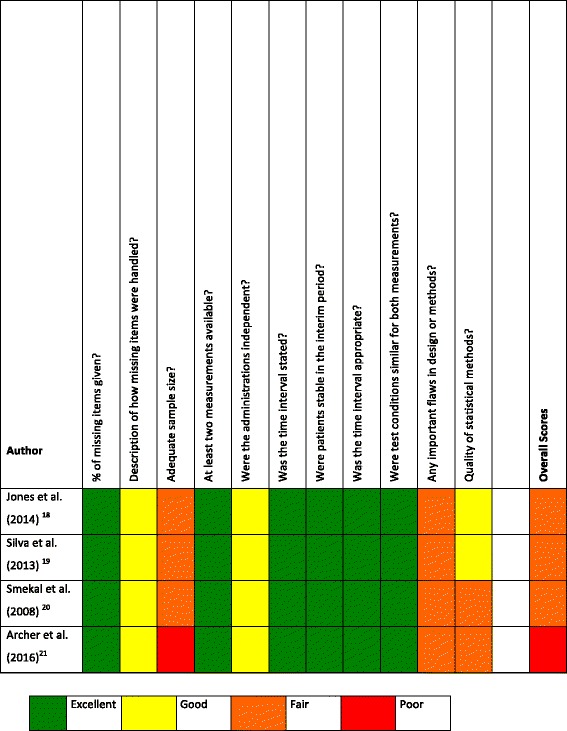
COSMIN checklist box B for assessment of reliability per included study per item

Using the 4-point-scale COSMIN checklist box B for assessment of reliability, three included studies were rated as fair and one as poor. The quality classification per study per item is described in Fig. 2.

### Studies included in the systematic review

Jones et al. [18] assessed the interrater and intrarater agreement for shortening and displacement using anterior-posterior (AP) and 30° caudo-cranial X-ray views in 30 patients. The measurements were performed by 13 observers on two occasions. The amount of shortening measured on radiograph was divided into seven categories: 0–5, 5.1–10.0, 10.1–15.0, 15.1–20.0, 20.1–25.0, 25.1–30, and > 30 mm. No to weak interrater agreement was found for shortening in the different categories. Displacement was divided into three categories: 0–49, 50–99, and 100%. Interrater agreement was minimal to weak. Intrarater agreement was moderate for displacement and minimal for shortening (Table 1).

Silva et al. [19] compared two methods of measuring shortening in 30 patients (32 fractures). The first was the method of choice of the observer, and the second was a standardized method. They used AP and 15° caudo-cranial views. Measurements were performed twice by seven observers. Intraclass correlation coefficients (ICC) with confidence intervals (CI) were calculated to determine interrater agreement, and average differences between the two time points with 95% CI were calculated to determine intrarater agreement.

For method 1, the interrater agreement was 0.771 (95% CI 0.655–0.865) and 0.743 (95% CI 0.604–0.851) at the two time points for fair agreement. The intrarater agreement for method 1 was 2.62 mm (95% CI 2.24–3.00) average difference between the two time points. For method 2, the interrater agreement was 0.741 (95% CI 0.629–0.842) and 0.685 (95% CI 0.554–0.805) at the two time points for fair and poor agreement, respectively. The intrarater agreement for method 2 was 3.34 mm (95% CI 2.88–3.80) average difference between the two time points.

Smekal et al. [20] assessed different modalities and views to determine the most accurate method compared to the CT in 30 patients. They used a standardized method of measuring. Measurements were performed by four observers on two occasions. A paired *t* test or a nonparametric Wilcoxon signed-rank test for determination of differences of mean values in paired samples was performed. The Kolmogorov-Smirnov test was used for determination of the distribution form. For the assessment of repeatability between occasions 1 and 2, the repeatability coefficient according to Bland and Altman was used. The differences among measurements on the four plain radiographs and CT scans were not significant. Also, there was no significant difference shown in measurements on both occasions. Repeatability coefficients were comparable for CT measurements, the posteroanterior thorax radiographs, and the 15° caudo-cranial anteroposterior panorama radiographs of the shoulder girdle. Repeatability coefficients for the clinical measurements and measurements on 15° caudo-cranial radiograph of the clavicle were markedly higher indicating lower repeatability.

Archer et al. [21] aimed to identify a correlation between plain AP film and computed tomography (CT) measurement of displacement and the inter- and intraobserver reliability of repeated radiographic measurements. Six observers (three orthopedic surgeons and three residents) measured the clavicles of 22 patients with an interval of 2 weeks. Shortening was assessed using the contralateral unfractured side as a reference. Participants were not instructed on what specific points within the fracture should be measured to estimate shortening and was therefore not standardized. The limits of agreement calculated using the Bland-Altman repeatability coefficient revealed a mean of ± 3.48 cm. The error inherent in plain film measurements in this study is 6.96 cm. Intraobserver agreement calculated with the paired *t* tests demonstrating a *p* > 0.05 in five of six observers. The authors conclude that plain AP film measurements of acute MSCF do not reliably predict shortening.

## Discussion

In this systematic review, we evaluated the reliability and reproducibility of measurements of shortening in MSCF. The results of this systematic review demonstrate that the literature on this topic did yield only three fair and one poor quality studies. Since shortening plays an increasingly important role in deciding on surgical intervention of MSCF, it is important to have a reliable and accurate method of measuring. Despite the lack of high-quality studies, the available knowledge and literature should not be discarded.

Smekal et al. [20] published a paper validating the accuracy/reliability of measurements of different imaging modalities and techniques. They found that the posterior-anterior (PA) thorax approximated the measurements on CT the best. Measurements on 15° tilted caudo-cranial radiograph of the clavicle and clinical measurements showed the smallest agreement with CT measurements. However, they did not state the reproducibility of measurements. The measurements were performed in healed malunited clavicle fractures and not in the acute phase. This was done to ensure static conditions in time. This is a strong feature of the study since Plocher et al. [22] described progressive shortening in acute MSCF in time.

The PA thorax means a higher dose for the patient of 0.1 mSv compared to 0.02 mSv for a clavicle AP [23]. It also relies on the symmetry of the clavicle using the unfractured side for comparison. A study by Cunningham et al. [24] reported asymmetry of the intact clavicle of more than 5 mm in almost 30% of patients. This may mean that measuring shortening of the MSCF compared to the unfractured side may be less reliable than assumed.

Archer et al. [21] also used the assumption of symmetry which may compromise reliability. They found a limit of agreement of 3.48 cm indicating that plain AP film of the fractured clavicle is not reliable in the prediction of the shortening measured on the CT scan. However, they found an ICC of 0.90. The statistical method for calculating intrarater variability using the paired *t* test may be debatable but they report no significant differences in measurements in five of six observers.

Jones et al. [18] reported weak to no agreement in inter- and intrarater agreement for radiological shortening using AP and 30° caudo-cranial views. They did not report a standardized method of measuring the shortening on these views. In addition, they also reported minimal to moderate interrater agreement for displacement and comminution. Intrarater agreement was strong for comminution, moderate for displacement, and minimal for shortening.

In contrast to current standard practice in which AP and 15° caudo-cranial views are made, papers have been published that support the use of a 15–30° cranio-caudal AP or PA or PA thorax view as being the most accurate in measuring the shortening of MSCF. [20, 25–27]. Although commenting on accuracy, these studies did not report the reproducibility of these views. Silva et al. [19] proposed a standardized mode of measuring shortening in MSCF. Their paper focused on adolescents, not adults, and also did not report the imaging modality or technique used. After contacting the corresponding author, it was verified that measurements were performed on standard AP and 15° caudo-cranial views. They reported no difference in a standardized measurement or method of choice concerning inter- and intraobserver variability. More recent studies find both a moderate and excellent interrater agreement using a standardized method of measuring [28, 29].

Two studies were not included in the review because these studies did not meet the inclusion criteria as only interrater agreement and not intrarater agreement was reported. However, we believe these studies are worth mentioning here. Stegeman et al. [29] found an intraclass correlation coefficient of 0.97 (CI 0.95–0.99) between two observers measuring shortening in a standardized way on 32 AP X-rays of the fractured clavicle. Interestingly, they found only a moderate agreement (0.45 CI 0.12–0.69) for measuring absolute shortening on the AP panoramic view after consolidation indicating that the imaging technique may be influential on the reliability of measurements as well. Malik et al. [28] report an ICC of 0.926 (CI 0.909–0.941) between four observers using a standardized method of measuring shortening of the fractured clavicle in 196 AP chest X-rays. These images were made with the patient varying between supine, semi-upright, and upright positions. The goal of this study was to evaluate differences in measured shortening between the different positions of the patients. No additional information on statistical analysis or interrater agreement per subgroup was reported.

Other factors reported to influence reliable and reproducible measurements are variation in magnification due to X-ray positioning and possibly positioning of the patient [18, 28, 30]. Backus et al. [30] reported a statistically significant difference between upright and supine patient positioning concerning shortening and displacement. Malik et al. [28] found a significant step-wise progression of measured shortening between supine, semi-upright, and upright positioning of the patient.

Some limitations of this study have to be discussed. First, there is only limited available literature on the topic of measuring the fractured clavicle. Since four studies were included and none of them were rated as good or excellent quality according to the COSMIN checklist, it was not possible to draw definite conclusions or make definite recommendations. Second, although the COSMIN checklist is considered the best available option to evaluate the methodological quality of studies on measurement properties, the “worst score counts” algorithm might underestimate the overall quality of a paper (e.g., one poor score out of a total of 11 items results in a poor overall score). For that reason, we provided the scores for all items using the 4-point scale. Other limitations of this study include the possibility of publication bias and language restrictions. Third, the inclusion criteria used might have been too strict. Two papers that did not meet the inclusion criteria were identified but yet could be of value on the topic. Including these papers [28, 29], however, does not influence the final conclusion pertaining the lack of evidence on the subject.

In order to optimize future studies and the realization of comparable results, a standardized method of imaging and measuring is of great importance. When considering the optimal method of imaging and measuring the fractured clavicle, one should consider the following: Imaging modality and technique, patient positioning, radiation exposure, costs and the method for measuring shortening, and/or displacement. To identify a standardized method, a compromise between these factors should be made based on further research.

CT scans and PA thorax seem more accurate, but the first is more expensive and both expose the patient to a much higher radiation dose. Supine positioning of the patient may underestimate the actual shortening and displacement, which in turn can negatively influence the decision to surgically reduce and fixate the MSCF. Calibrated views will prevent magnification errors while measuring. Although not proven better, it might be a consideration to optimize consistency by measuring shortening and displacement in a standardized and possibly proportional way as proposed by other authors. [9, 13, 19, 30, 31]

## Conclusion

The objective of this systematic review was to evaluate the reliability and reproducibility of measurements of shortening in MSCF using any available imaging technique.

We identified four studies on reliability of measurement of MSCF. Since these studies were only of fair and poor quality, it was impossible to draw definite conclusions. Shortening is one of the reasons to surgically treat the fractured clavicle, so further research is needed to identify the most effective, reproducible, and reliable method of imaging and measuring. In order to optimize future studies and the realization of comparable results, a standardized method of imaging and measuring is of great importance.

## Additional files

Additional file 1: Search query PubMed, EMBASE, and Cochrane. (DOCX 24 kb)

Additional file 2: Nonregistered PROSPERO review protocol. ReviewProtocol_MeasurementsClavicle. (PDF 60 kb)

Additional file 3: PRISMA 2009 checklist. PRISMA 2009 checklist. (PDF 65 kb)

## Abbreviations

AP: Anterior-posterior
CI: Confidence interval
CT: Computed tomography
ICC: Intraclass correlation coefficient
MSCF: Midshaft clavicle fractures
PA: Posterior-anterior
PRISMA: Preferred Reporting Items for Systematic Reviews and Meta-Analyses
