# Supplementary material for: Reliability of measurements of the fractured clavicle: a systematic review
Source: Syst Rev. 2017 Nov 3;6:223. doi: 10.1186/s13643-017-0614-4 (PMC5670711; doi:10.1186/s13643-017-0614-4)
Supplement: Supplementary file 1 — Search query PubMed, EMBASE, and Cochrane. (DOCX 24 kb) [file 13643_2017_614_MOESM1_ESM.docx]

**Additional file 1**

*Pubmed*

((clavic OR clavical OR clavicle OR clavicle' OR clavicle'' OR clavicle's OR claviclefractures OR clavicles OR clavicles' OR claviclular OR clavicula OR claviculae OR clavicular OR (collar AND bone) OR (collar AND bones) OR claviculas))

AND

(fracture OR fracture' OR fracture's OR fractures OR fractures')

AND

(length OR length' OR lengthening OR lengthening' OR lengthens OR lengths OR lengths' OR lengthscale OR lengthscales)

(shorten OR shortened OR shortened' OR shortening OR shortening' OR shortenings OR shortens)

(displace OR displaced OR displaced' OR displaceing OR displacement OR displacement' OR displacements OR displaces)

AND

(radiologic OR radiological OR radiological' OR radiologically OR radiologische OR radiologist OR radiologist' OR radiologist's OR radiologists OR radiologists' OR radiology OR radiology' OR radiology's)

(radiograph OR radiographs OR radiographical OR radiograph’)

(image OR images OR imaging OR imagings)

(X-ray OR x-rays OR x-ray’s OR (X AND ray) OR (x AND rays))

AND

(Repeatability OR repeatibilities OR repeatable)

(measure OR measure' OR measure's OR measureable OR measureables OR measureably OR measured OR measured' OR measurements OR measurements' OR measurer OR measurer' OR measurer's OR measurers OR measurers' OR measures OR measures')

(reliabilities OR reliability OR reliability' OR reliable OR reliable')

(reproduce OR reproduce' OR reproduced OR reproduced' OR reproducibility OR reproducibility OR reproducible OR reproducible' OR reproducing OR reproducing')

((observer OR observer' OR observers OR observers' OR interobserver OR interobserver's OR interobservers OR interobservers' OR intraobserver OR intraobserver's OR intraobservers OR (intra AND observer) OR (intra AND observer) OR inter-observer OR intra-observer OR interrater OR rater OR intrarater OR (intra AND rater) OR (intra AND rater ) OR inter-rater OR intra-rater))

(valid OR validity OR validate OR validate' OR validated OR validated' OR validating OR validating' OR validation OR validation')

(variability OR variability' OR variability's OR variable OR variable' OR variables OR variables')

(accuracy OR accuracy' OR accurate OR accurately OR accurateness)

*Embase*

((clavic OR clavical OR clavicle OR clavicle' OR clavicle'' OR clavicle's OR claviclefractures OR clavicles OR clavicles' OR claviclular OR clavicula OR claviculae OR clavicular OR (collar AND bone) OR (collar AND bones) OR claviculas)).af

AND

(fracture OR fracture' OR fracture's OR fractures OR fractures').af

AND

(length OR length' OR lengthening OR lengthening' OR lengthens OR lengths OR lengths' OR lengthscale OR lengthscales).af

(shorten OR shortened OR shortened' OR shortening OR shortening' OR shortenings OR shortens).af

(displace OR displaced OR displaced' OR displaceing OR displacement OR displacement' OR displacements OR displaces).af

AND

(radiologic OR radiological OR radiological' OR radiologically OR radiologische OR radiologist OR radiologist' OR radiologist's OR radiologists OR radiologists' OR radiology OR radiology' OR radiology's).af

(radiograph OR radiographs OR radiographical OR radiograph).af

(image OR images OR imaging OR imagings).af

(X-ray OR x-rays OR (X AND ray) OR (x AND rays)).af

AND

(Repeatability OR repeatibilities OR repeatable).af

(measure OR measure' OR measure's OR measureable OR measureables OR measureably OR measured OR measured' OR measurements OR measurements' OR measurer OR measurer' OR measurer's OR measurers OR measurers' OR measures OR measures').af

(reliabilities OR reliability OR reliable).af

(reproduce OR reproduce OR reproduced OR reproduced' OR reproducibility OR reproducibility OR reproducible OR reproducing).af

((observer OR observer' OR observers OR observers' OR interobserver OR interobserver's OR interobservers OR interobservers' OR intraobserver OR intraobserver's OR intraobservers OR (intra AND observer) OR (intra AND observer) OR inter-observer OR intra-observer OR interrater OR rater OR intrarater OR (intra AND rater) OR (intra AND rater ) OR inter-rater OR intra-rater)).af

(valid OR validity OR validate OR validate' OR validated OR validated' OR validating OR validating' OR validation OR validation').af

(variability OR variability' OR variability's OR variable OR variable' OR variables OR variables').af

(accuracy OR accuracy' OR accurate OR accurately OR accurateness).af
