# Supplementary material for: Reliability of measurements of the fractured clavicle: a systematic review
Source: Syst Rev. 2017 Nov 3;6:223. doi: 10.1186/s13643-017-0614-4 (PMC5670711; doi:10.1186/s13643-017-0614-4)
Supplement: Supplementary file 2 — Nonregistered PROSPERO review protocol. ReviewProtocol_MeasurementsClavicle. (PDF 60 kb) [file 13643_2017_614_MOESM2_ESM.pdf]

Review title and timescale

1 Review title

Reliability of measurements of the fractured clavicle. A systematic review.

2 Original language title

English, Dutch

3 Anticipated or actual start date

07/01/2014

4 Anticipated completion date

03/01/2015

5 Stage of review at time of this submission

Indicate the stage of progress of the review by ticking the relevant boxes. Reviews that have progressed beyond the point of completing data extraction at the time of initial registration are not eligible for inclusion in PROSPERO. This field should be updated when any amendments are made to a published record.

The review has not yet started ✖

Review stage	Started	Completed
Preliminary searches	Yes	Yes
Piloting of the study selection process	Yes	Yes
Formal screening of search results against eligibility criteria	Yes	Yes
Data extraction	Yes	Yes
Risk of bias (quality) assessment	Yes	Yes
Data analysis	Yes	Yes

Provide any other relevant information about the stage of the review here.

Review team details

6 Named contact

The named contact acts as the guarantor for the accuracy of the information presented in the register record.

Paul Hoogervorst

7 Named contact email

Enter the electronic mail address of the named contact.

Paul.hoogervorst@radboudumc.nl

8 Named contact address

Enter the full postal address for the named contact.

Department of Orthopaedics, Radboud university medical center
P.O. Box 9101, 6500 HB Nijmegen, the Netherlands

9 Named contact phone number

Enter the telephone number for the named contact, including international dialing code.

0031 24 36 14148

10 Organizational affiliation of the review

Full title of the organizational affiliations for this review, and website address if available. This field may be completed as 'None' if the review is not affiliated to any organization.

Department of Orthopaedics, Radboud university medical center, Nijmegen, the Netherlands

Website address: https://www.radboudumc.nl/

11 Review team members and their organizational affiliations

Give the title, first name and last name of all members of the team working directly on the review. Give the organisational affiliations of each member of the review team.

Title	First name	Last name	Affiliation
Mr	Paul	Hoogervorst	Department of Orthopaedics, Radboudumc
Mr	Gerjon	Hannink	Department of Orthopaedics, Radboudumc
Mr	Arnoud	Van Geene	Department of Orthopaedics, OLVG
Mr	Albert	Van Kampen	Department of Orthopaedics, Radboudumc

12 Funding sources/sponsors

Give details of the individuals, organizations, groups or other legal entities who take responsibility for initiating, managing, sponsoring and/or financing the review. Any unique identification numbers assigned to the review by the individuals or bodies listed should be included.

N/A

13 **Conflicts of interest**
List any conditions that could lead to actual or perceived undue influence on judgements concerning the main topic investigated in the review.
None
Are there any actual or potential conflicts of interest?
No

14 **Collaborators**
Give the name, affiliation and role of any individuals or organizations who are working on the review but who are not listed as review team members.

Title	First name	Last name	Organisation details

Review methods

15 **Review question(s)**
State the question(s) to be addressed / review objectives. Please complete a separate box for each question.
The objective of this systematic review is to evaluate the reliability and reproducibility of measurements of shortening in MSCF using any available imaging technique.

16 **Searches**
Give details of the sources to be searched, and any restrictions (e.g. language or publication period). The full search strategy is not required, but may be supplied as a link or attachment.
Search Electronic databases (PubMed, EMBASE and Cochrane) from their inception to November 2016. Use the search strategy as described in Appendix 1.

17 **URL to search strategy**
If you have one, give the link to your search strategy here. Alternatively you can e-mail this to PROSPERO and we will store and link to it.
I give permission for this file to be made publicly available

18 **Condition or domain being studied**
Give a short description of the disease, condition or healthcare domain being studied. This could include health and wellbeing outcomes.
Measurements of shortening in the fractured clavicle

19 **Participants/population**
Give summary criteria for the participants or populations being studied by the review. The preferred format includes details of both inclusion and exclusion criteria.
Studies aiming to assess shortening of the fractured clavicle for intrarater and interrater reliability.

• Studies investigating methods of imaging of the fractured clavicle for intrarater and interrater reliability.

• Only original studies were included.

• Studies in Dutch or English.

• Study population aged 9 years and older

20 **Intervention(s), exposure(s)**
Give full and clear descriptions of the nature of the interventions or the exposures to be reviewed
N/A

21 **Comparator(s)/control**
Where relevant, give details of the alternatives against which the main subject/topic of the review will be compared (e.g. another intervention or a non-exposed control group).
N/A

22 **Types of study to be included initially**
Give details of the study designs to be included in the review. If there are no restrictions on the types of study design eligible for inclusion, this should be stated.
All types of study design except abstracts, theses or conference proceedings.

23 **Context**
Give summary details of the setting and other relevant characteristics which help define the inclusion or exclusion criteria.

24 **Primary outcome(s)**
Give the most important outcomes.
Give information on timing and effect measures, as appropriate.
intrarater and interrater reliability for assessing shortening of the fractured clavicle.

intrarater and interrater reliability for methods of imaging of the fractured clavicle.

25 **Secondary outcomes**

List any additional outcomes that will be addressed. If there are no secondary outcomes enter None.

Give information on timing and effect measures, as appropriate.

None

26 **Data extraction, (selection and coding)**

Give the procedure for selecting studies for the review and extracting data, including the number of researchers involved and how discrepancies will be resolved. List the data to be extracted.

Extract data from all included studies with respect to specific characteristics. These are number of clavicles reviewed, study design, imaging technique, method of measurements, statistical analysis, and the authors conclusion.

PH and GH will extract data independently. If disagreement persisted after discussion, consensus will be reached after consulting AvK.

27 **Risk of bias (quality) assessment**

State whether and how risk of bias will be assessed, how the quality of individual studies will be assessed, and whether and how this will influence the planned synthesis.

Methods and quality are independently assessed (PH and GH, any discrepancies were discussed to achieve consensus, using a third reviewer (AvK) for all included studies.

28 **Strategy for data synthesis**

Give the planned general approach to be used, for example whether the data to be used will be aggregate or at the level of individual participants, and whether a quantitative or narrative (descriptive) synthesis is planned. Where appropriate a brief outline of analytic approach should be given.

A descriptive analysis is planned

29 **Analysis of subgroups or subsets**

Give any planned exploration of subgroups or subsets within the review. ‘None planned’ is a valid response if no subgroup analyses are planned.

None planned

Review general information

30 **Type of review**

Select the type of review from the drop down list.

Systematic Review

31 **Language**

Select the language(s) in which the review is being written and will be made available, from the drop down list. Use the control key to select more than one language.

Will a summary/abstract be made available in English?

English

32 **Country**

Select the country in which the review is being carried out from the drop down list. For multi-national collaborations select all the countries involved. Use the control key to select more than one country.

The Netherlands

33 **Other registration details**

Give the name of any organization where the systematic review title or protocol is registered together with any unique identification number assigned. If extracted data will be stored and made available through a repository such as the Systematic Review Data Repository (SRDR), details and a link should be included here.

N/A

34 **Reference and/or URL for published protocol**

Give the citation for the published protocol, if there is one.

Give the link to the published protocol, if there is one. This may be to an external site or to a protocol deposited with CRD in pdf format.

I give permission for this file to be made publicly available

35 **Dissemination plans**

Give brief details of plans for communicating essential messages from the review to the appropriate audiences.

Disseminate results in a peer-review journal

Do you intend to publish the review on completion?

Yes

36 **Keywords**

Give words or phrases that best describe the review. (One word per box, create a new box for each term)

37 **Details of any existing review of the same topic by the same authors**

Give details of earlier versions of the systematic review if an update of an existing review is being registered, including full bibliographic reference if possible.
No

38 Current review status

Review status should be updated when the review is completed and when it is published.

39 Any additional information

Provide any further information the review team consider relevant to the registration of the review.
N/A

40 Details of final report/publication(s)

This field should be left empty until details of the completed review are available.
Give the full citation for the final report or publication of the systematic review.
Give the URL where available.
